# Parliament and the Profession

**Published:** 1918-05-11

**Authors:** 


### PARLIAMENT AND THE PROFESSION.

### Women Entitled to Wear the Chevron.

MR. MACPHERSON stated that the Regulations provide for the award of the overseas chevron to all women employed under authority with any of the British Forces outside the United Kingdom. The several categories that may be awarded chevrons include: Members of Nursing Services, of Queen Mary's Army Auxiliary Corps, of Voluntary Aid Detachments, and of the Women's Legions. *Personnel* working under the Joint War Committee of the British Red Cross Society and the Order of St. John of Jerusalem, and organisations affiliated thereto, and the St. Andrew's Ambulance Association, and women holding official appointments with the British Forces are also eligible.

### Future of the Red Cross and Order of St. John.

THE House of Lords has passed a Bill entitled "An Act to enable the British Red Cross Society and the Grand Priory of the Order of St. John of Jerusalem in England to apply to the public advantage the residue of their property acquired for the purposes of the present war and for other purposes."

### Soldiers with Venereal Disease.

MR. MACPHERSON stated that officers admitted to hospital with venereal disease are required to pay the full hospital stoppage—namely, 2s. 6d. a day—and forfeit their field allowance and lodging, fuel, and light allowances. Soldiers are required to pay hospital stoppages of 7d. a day and forfeit proficiency pay, corps or engineer pay, and the increases of pay recently authorised. Concealment of venereal disease is dealt with under the Army Act.

### Medical Students and Military Service.

IN reply to a question addressed by Mr. Snowden to the Minister of National Service, Mr. Beck stated that fit men who were on March 5, 1918, full-time medical students at a recognised medical school, and who either had already passed the whole of the first professional examination in chemistry, physics, and biology (or botany and zoology), or who are certified as being able to pass the whole of that examination on or before July 31, 1918, are exempted from military service under certain conditions—firstly, as to the efficient prosecution of their studies, and, secondly, as to training in an officers' training corps. On completing their second examination, such students, if physically fit, are required to serve as surgeon-probationers in the Navy.

Students in Grade III., subject to similar provisions as to work and training, are not to be called up without reference to the Minister of National Service so long as they remain full-time students.

Men serving with the Colours who had immediately prior to enlistment been whole-time students at a recognised medical school for at least six months and had passed the whole of the first professional examination may, on application, be released to resume their studies. Medical students who do not fulfil the conditions of exemption are posted to the Army in the ordinary way, and not necessarily to the R.A.M.C.

